# TaPIMP2, a pathogen-induced MYB protein in wheat, contributes to host resistance to common root rot caused by *Bipolaris sorokiniana*

**DOI:** 10.1038/s41598-017-01918-7

**Published:** 2017-05-11

**Authors:** Xuening Wei, Tianlei Shan, Yantao Hong, Huijun Xu, Xin Liu, Zengyan Zhang

**Affiliations:** The National Key Facility for Crop Gene Resources and Genetic Improvement, Institute of Crop Science, Chinese Academy of Agricultural Sciences, Beijing, 100081 China

## Abstract

MYB transcription factors (TFs) have been implicated in various biology processes in model plants. However, functions of the great majority of MYB TFs in wheat (*Triticum aestivum* L.) have not been characterized. The soil-borne fungal pathogens *Bipolaris sorokiniana* and *Rhizoctonia cerealis* are the causal agents of important destructive diseases of wheat. Here, the *TaPIMP2* gene, encoding a pathogen-induced MYB protein in wheat, was isolated through comparative transcriptomic analysis, and its defensive role was studied. TaPIMP2 was proved to localize in nuclei. *TaPIMP2* responded in a different extent and speed upon infections of *B*. *sorokiniana* or *R*. *cerealis*. *TaPIMP2* displayed different expression patterns after exogenous application of phytohormones, including abscisic acid, ethylene, and salicylic acid. Silencing of *TaPIMP2* repressed resistance of wheat cultivar Yangmai 6 to *B*. *sorokiniana*, but did not alter resistance of wheat line CI12633 to *R*. *cerealis*. *TaPIMP2* overexpression significantly improved resistance to *B*. *sorokiniana* rather than *R*. *cerealis* in transgenic wheat. Moreover, *TaPIMP2* positively modulated the expression of pathogenesis-related genes, including *PR1a*, *PR2*, *PR5*, and *PR10*. Collectively, *TaPIMP2* positively contributes to wheat resistance to *B*. *sorokiniana* possibly through regulating the expression of defense-related genes, and *TaPIMP2* plays distinct roles in defense responses to different fungal infection.

## Introduction

Plants possess an elaborate and complex immune system to recognize and respond to pathogen infection^1^. Major progress has been made towards the understanding of plant immune system. Numerous defense-related events, including pathogen perception, signal generation and transmission, and activation of defense products restricting pathogen invasion, have been identified. Perception of pathogens by host plants induces robust and selective transcriptional reprogramming that is central for triggering effective defense response to invading pathogens. Phytohormones, such as salicylic acid (SA), jasmonic acid (JA), ethylene, and abscisic acid (ABA), plays pivotal roles in regulation of defense signaling network^2^. Accumulating evidence indicates that transcription factors (TFs) play important regulatory roles in plant defense response to biotic stress^3, 4^.

Myeloblastosis (MYB) TFs belong to one of the largest TF families^5, 6^. MYB TFs contain one to four highly conserved DNA-binding MYB domains, and can be divided into 4 major subgroups, 1R-MYB (MYB-related), R2R3-MYB, 3R-MYB, and 4R-MYB^6^. The MYB TFs have been studied in certain plant species, such as *Arabidopsis thaliana*, maize (*Zea mays*), rice (*Oryza sativa*), grapevine (*Vitis vinifera*), poplar (*Populus tremuloides*), apple (*Malus domestica*), and wheat (*Triticum aestivum*). MYB proteins have been confirmed to play crucial regulation roles in diverse physiological and biological processes, including cell cycle regulation, cell wall biosynthesis, development and reproduction, hormonal signaling, primary and secondary metabolism, and tolerance to abiotic stress^7–14^. Some MYB TFs participate in plant defense responses to invading pathogens^7, 15–20^. For example, two R2R3-MYB proteins from Arabidopsis, BOS1 (BOTRYTIS-SUSCEPTIBLE1, AtMYB108)^7^ and AtMYB96^15^, are required for defense responses to necrotrophic pathogens (*Botrytis cinerea* and *Alternaria brassicicola*) and to the bacteria pathogen *Pseudomonas syringae* DC3000, respectively. Overexpression of *TiMYB2R*-*1*, an R2R3-MYB gene in *Thinopyrum intermedium*, significantly enhanced resistance of the transgenic wheat to take-all disease caused by *Gaeumannomyces graminis* ^16^. In barley, an MYB TF HvMYB6 positively regulates basal resistance and MLA-mediated immunity response to *Blumeria graminis* ^17^. In wheat, TaPIMP1, the pathogen-induced MYB protein1, positively contributes host resistance to infection of the fungal pathogen *Bipolaris sorokiniana* and drought stresses^18^. Silencing of the R2R3-MYB gene *TaMYB4* and the MYB1R gene *TaLHY* increased susceptibility to the biotrophic fungal pathogen *Puccinia striiformis* f. sp. *Tritici* (*Pst*) in wheat^19, 20^, respectively. However, functional roles of the great majority of MYB TFs have not yet been investigated in plant species.

Wheat is one of most important staple crops and plays a fundamental role in food security worldwide. Wheat production was often negatively affected by the infection of a wide variety of pathogens. Common root rot is one of the most serious wheat diseases worldwide. *Bipolaris sorokiniana*, a soil-borne fungal pathogen, is the causal agent of common root rot. *B*. *sorokiniana* also can cause leaf spot blotch, seedling blight, head blight, and black point of grains in wheat and barley, and other species of cereals and grasses^21, 22^. Additionally, the sharp eyespot, mainly caused by the necrotrophic fungus *Rhizoctonia cerealis*, is a destructive disease that seriously reduces wheat grain yield in some regions of the world^23^. For example, in China, since 2005 to date, wheat plants in at least 8 million ha have been subjected to sharp eyespot disease, resulting in yield losses of 10~30% (http://www.agri.cn/V20/bchqb/201501/t20150121_4344729.htm). The efficient and environmentally-safe means to protect wheat from *B*. *sorokiniana* and *R*. *cerealis* infection is to develop disease-resistant varieties. The efficiency of breeding depends on the availability of resistant germplasm and on an understanding the wheat defense responses. However, traditional breeding of resistant wheat varieties is difficult since no completely-resistant wheat accessions have been available. Previous studies^18–20, 24^ reported that 4 wheat MYB genes, including *TaPIMP1*, *TaMYB4*, *TaLHY*, *and TaRIM1*, positively participate in infection of the soil-borne fungal pathogen *B*. *sorokiniana*, the biotrophic fungal pathogen *Pst*, and the necrotrophic pathogen *R*. *cerealis*, respectively. Genetic engineering of resistance-related MYB TFs has been proposed to be an important strategy for improving the disease resistance of wheat. Therefore, it is vital to identify important resistance-related MYB genes and to dissect their functions.

In this study, an MYB gene was identified that displayed the differential induction between the resistant CI12633 and susceptible Wenmai 6 upon inoculation with *R*. *cerealis* based on the wheat microarray data obtained previously by our lab (GEO accession number GSE69245)^25^. The transcription of this MYB gene was also up-regulated in *B*. *sorokiniana*-resistant wheat Yangmai 6 after infection of *B*. *sorokiniana*. In a previous study, the wheat MYB gene *TaPIMP1* was characterized, which contributes to host resistance to *B*. *sorokiniana* infection and drought tolerance^18^. Thus, the MYB gene characterized in this study was designated as *TaPIMP2*. *TaPIMP2* exhibited distinct expression patterns in responses to the infections of *B*. *sorakiniana* and *R*. *cerealis*. The defense functions of *TaPIMP2* to these two pathogens were investigated through generation and assessment of *TaPIMP2* knockdown and overexpression wheat plants. The alteration of expression level of *TaPIMP2* affected the wheat resistance degree to *B*. *sorokiniana* infection rather than *R*. *cerealis*. These results suggested that *TaPIMP2* plays distinct roles in different wheat responses to the infection of *B*. *sorokiniana* or *R*. *cerealis*.

## Results

### Isolation and sequence analyses of *TaPIMP2* gene

Through Agilent Wheat GeneChip-based transcriptomic analyses, we identified 1,533 genes (GEO accession number GSE69245) that were expressed in more than 2-fold higher level in *R*. *cerealis*-resistant wheat line CI12633 than in *R*. *cerealis*-susceptible wheat cultivar Wenmai 6 following 4 and 21 day post inoculation (dpi) with *R*. *cerealis* isolate R0301^25^. Among them, a probe with the number A_99_P129195, corresponding to the 3′-end sequence of a wheat MYB cDNA (GenBank accession no. AB252145), was ﻿﻿selected﻿ to﻿ be﻿ characterized. Its transcript levels in CI12633 increased 2.854-fold at 4 dpi and 5.204-fold at 21 dpi than that in Wenmai 6. The wheat gene corresponding to the probe A_99_P129195 was named *TaPIMP2* (pathogen-induced MYB protein 2 in *Triticum aestivum*). The full-length cDNA sequence (1234 bp) of *TaPIMP2* was obtained through 2 round 3′-RACE PCRs from *R*. *cerealis*-infected stem cDNA of CI12633 and deposited in the GenBank database (GenBank accession no. KX683396), as well as can be cloned from *B*. *sorokiniana* -infected stem cDNA of the wheat line Yangmai 6. The genomic DNA sequence of *TaPIMP2* was also amplified from CI12633. Comparison of the genomic DNA and cDNA sequence revealed that the *TaPIMP2* gene structure includes two exons and an intron with 141 nucleotides. The cDNA sequence of *TaPIMP2* includes an ORF with 882 nucleotides (from no. 67 to 948 bp). The ORF sequence of *TaPIMP2* shares a 44.51% identity with *TaRIM1*.

**Figure 1:**
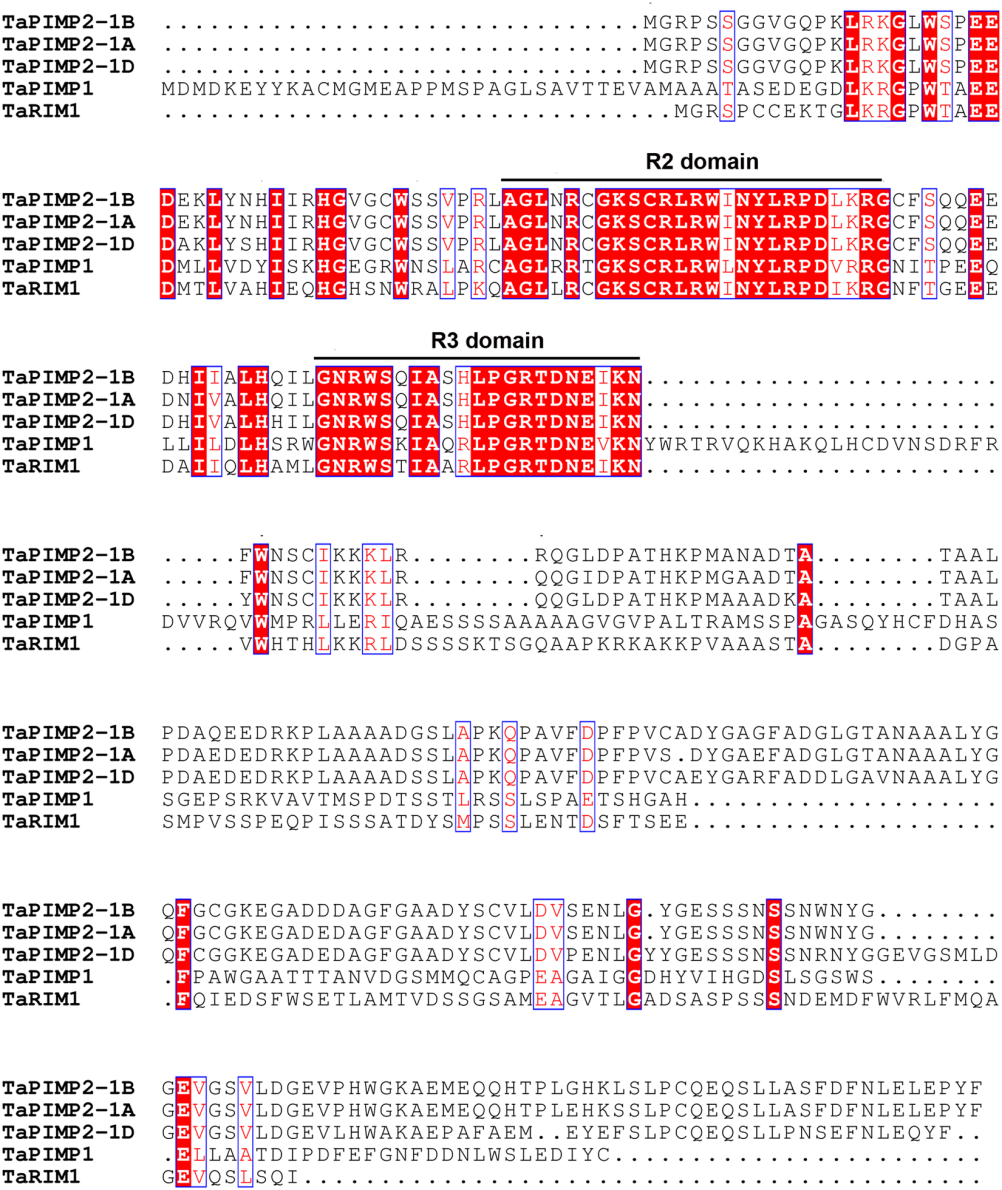
Multiple sequence alignment of TaPIMP2, TaRIM1, and TaPIMP1 from wheat. The R2 and R3 domains are indicate by solid lines.

**Figure 2:**
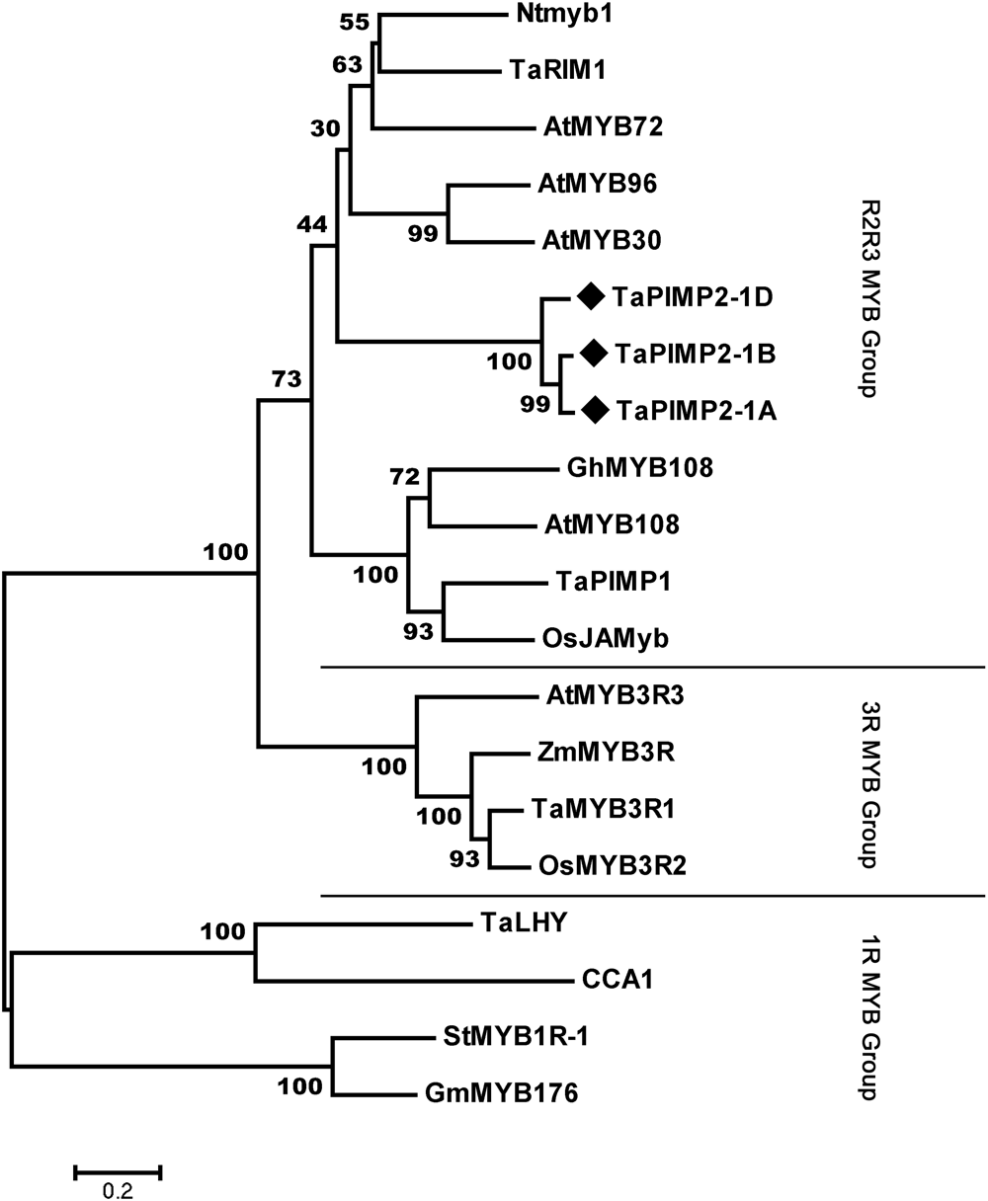
A phylogenetic analysis of 18 known-functional MYB members from 8 plant species. TaPIMP2 from wheat was indicated by black box. The Genbank accession number of proteins including this analysis: Ntmyb1: AAB41101; TaRIM1: AMP18876; AtMYB96: AED97610; AtMYB30: Q9SCU7; AtMYB72: NP_176012; GhMYB108: ALL53614; AtMYB108: Q9LDE1; TaPIMP1: ABU93236; OsJAMyb: AAK08983; AtMYB3R3: NP_001078127; ZmMYB3R3: NP_001151448; TaMYB3R1: ADO32617; OsMYB3R2: NP_001044767; StMYB1R-1: NP_001275346; GmMYB176: ABH02865; TaLHY: ADW09013; CCA1: AEC10760.

The deduced protein TaPIMP2 is consisted of 293 amino acids, and contains two conserved MYB domains (Fig. 1). In previous studies from our laboratory we characterized two MYB genes, *TaPIMP1* ^18^ and *TaRIM1* ^24^, which were involved into wheat resistance to *B*. *sorokiniana* and *R*. *cerealis*, respectively. Here, the putative protein sequence of TaPIMP2 was aligned with TaPIMP1 and TaRIM1 (Fig. 1). The result showed that two MYB domains [one (R2) located at amino acids 15–65 and other one (R3) at 68–116] were conserved among these 3 MYB proteins. Except the conserved MYB domains, whole amino acid sequences of these 3 proteins exhibited a low sequence similarity and identity. The whole protein sequence of TaPIMP2 shared 35.0% sequence identity with TaRIM1 (GenBank accession no. KU864997) and 26.9% sequence identity with TaPIMP1 (GenBank accession no. EF587267), respectively. In the reconstructed phylogenetic tree including the 18 known-functional MYB proteins from 8 plant species, TaPIMP2 was clustered into the R2R3-MYB subgroup (Fig. 2). The phylogenetic tree analysis showed that TaPIMP2 was the closest to AtMYB72 from Arabidopsis, while TaPIMP1 and TaRIM1, other defense-related wheat MYB proteins, were clustered into different clades in R2R3-MYB subgroup, respectively. These results indicated that TaPIMP2 is a R2R3 MYB protein and might be involved in wheat defense responses.

### Expression patterns of *TaPIMP2* in wheat challenging with *R*. *cerealis* and *B*. *sorokiniana*

Hexaploid wheat (AABBDD) contains three sub-genomes. Here, the expression profiles of *TaPIMP2* homoeologous members from the 3 differential sub-genomes were investigated. Using nucleotide acid sequence of *TaPIMP2* as query to blast wheat survey sequences database (https://urgi.versailles.inra.fr/blast), we obtained three homoeologous members that displayed highly sequence identity with *TaPIMP2*. The three sequences were TRIAE_CS42_1AL_TGACv1_002296, TRIAE_CS42_1BL_TGACv1_030624, and TRIAE_CS42_1DL_TGACv1_062692. *TaPIMP2* displayed the highest sequence identity (99.9%) with TRIAE_CS42_1BL_TGACv1_030624. These results implied that *TaPIMP2* have three homoeologous members in hexaploid wheat, which were derived from 1A, 1B, and 1D subgroup, respectively. The three homoeologous members were designated as *TaPIMP2*-*1A*, *TaPIMP2*-*1B*, and*TaPIMP2*-*1D*. The homoeologous member-specific primers were designed for detecting the transcriptions of the homoeologous genes.

**Figure 3:**
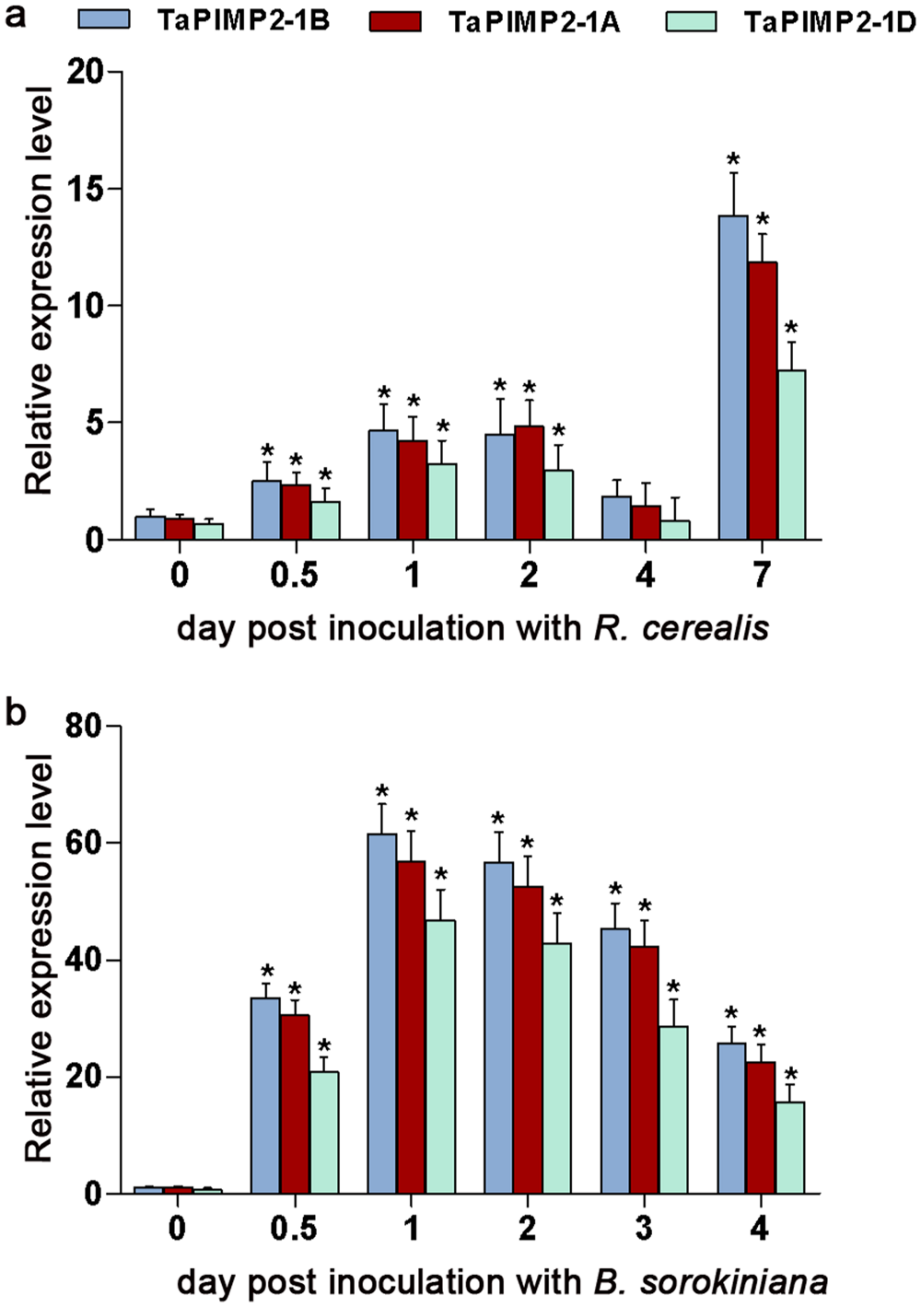
Transcriptional patterns of *TaPIMP*2 homoeologous member response to fungal infection. Transcription of *TaPIMP*2 homoeologous member response to infection with *Rhizoctonia cerealis*in in wheat line CI12633 (**a**) and infection with *Bipolaris sorokiniana* in wheat cultivar Yangmai 6 (**b**) was examined by RT-qPCR. The samples were collected from stem of infected wheat plants. The relative gene expression was quantified using the comparative threshold (2^−ΔΔCT^) method. The transcriptional level of *TaPIMP2*-*1B* from the untreated wheat plants was used as control. The mean and standard deviation were calculated using data from three independent biological replicates. Asterisks indicate statistically significant differences. (Student’s *t*-*test*: *P < 0.05).

The transcriptional patterns of *TaPIMP2* homoeologous members in wheat stems defense to *R*. *cerealis* or *B*. *sorokiniana* were investigated by RT-qPCR. As shown in Fig. 3a and b, after pathogens infection, three homoeologous members exhibited similar expression trends. *TaPIMP2*-*1B* and *TaPIMP2*-*1D* possessed similar transcriptional levels, while *TaPIMP2*-*1A* had a slightly lower transcriptional level than the former two members. Compared with the untreated wheat plants, the transcription of *TaPIMP2* in *R*. *cerealis*-resistant wheat line CI12633 stems was gradually elevated after inoculated with *R*. *cerealis*. The transcriptional level reached to ~4-fold at both 1 dpi (day post inoculation) and 2 dpi, subsequently decreased (at 4 dpi similar to that in untreated plants), and increased to the peak at 7 dpi (~14-fold). In addition, the expression of *TaPIMP2* was measured in wheat cultivar Yangmai 6 after inoculation with *B*. *Sorokiniana*. Wheat cultivar Yangmai 6 exhibited resistance to infection with *B*. *sorokiniana*. Upon *B*. *sorokiniana* infection, transcriptional accumulation of *TaPIMP2* rapidly and dramatically increased ~60-fold at 1 dpi compared with untreated plants, then gradually decreased at following days (2, 3, and 4 dpi), with ~25-fold at 4 dpi compared with untreated plants (Fig. 3b). This distinct expression profiles of *TaPIMP2* in wheat defense responses against the above two different fungal pathogens suggested that *TaPIMP2* may be involved in defense responses to infection of *B*. *sorokiniana* or *R*. *cerealis* in a different extent.

### *TaPIMP2* transcriptional responses to exogenous hormones in wheat plants

**Figure 4:**
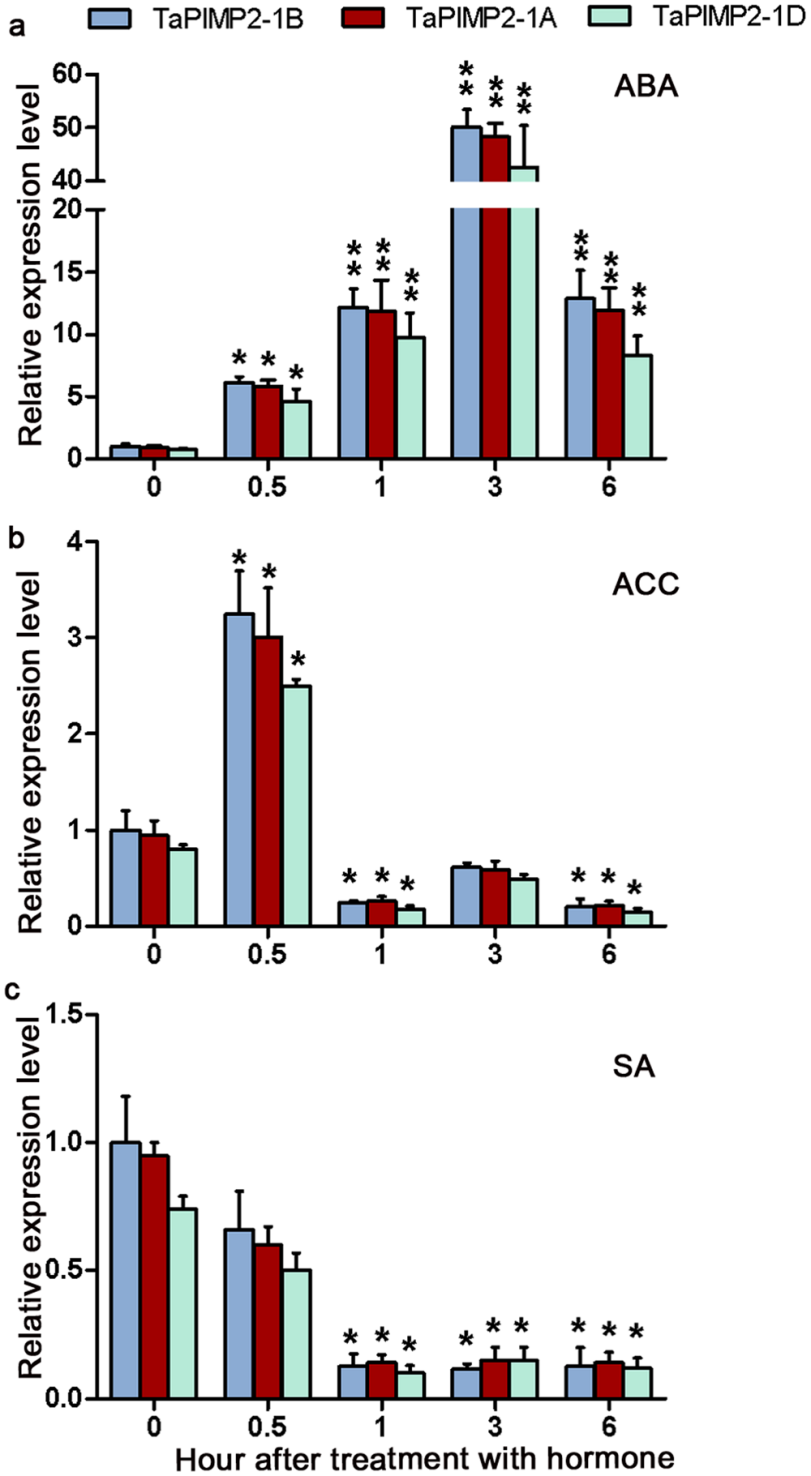
Transcriptional patterns of *TaPIMP*2 homoeologous members after treatment with hormones. The wheat cultivar Yangmai 16 plants were sprayed with abscisic acid (ABA), 1-aminocyclopropane-1-carboxylic acid (ACC, precursor of ethylene), salycilic acid (SA), or Tween-20 solution. The transcriptional level of *TaPIMP2*-*1B* from the wheat plants treated with Tween-20 was used as control. The mean and standard deviation were calculated using data from three independent biological replicates. Asterisks indicate statistically significant differences. (Student’s *t*-*test*: *P < 0.05, **P < 0.01).

Transcriptional profiles of *TaPIMP2* homoeologous members, *TaPIMP2-1A*, -*1B*, and -*1D* were analyzed by qPCR in Yangmai16 leaves after treatments with exogenous defense-related hormones ABA, 1-aminocyclopropane-1-carboxylic acid (ACC, precursor of ethylene), and SA. As shown in Fig. 4a, *TaPIMP2* expression levels were gradually induced by ABA. The accumulation reached a peak at 3 hour after the treatment, increased ~50 fold compared with control plants. After treatment with ACC, transcription of *TaPIMP2* was increased to ~3 fold at 0.5 hour, subsequently decreased to 0.5 fold at 1, 3, and 6 hour compared with control plants (Fig. 4b). However, transcription of *TaPIMP2* was repressed by exogenous SA, which showed a reduction to 0.2-fold of control plants at 1, 3, and 6 hour after treatment (Fig. 4c). *TaPIMP2* homoeologous members shared similar expression tendency, and the accumulation of *TaPIMP2*-*1D* was slightly lower than those of *TaPIMP2*-*1B* and -*1A*.

### TaPIMP2 is a nuclear-expression protein

**Figure 5:**
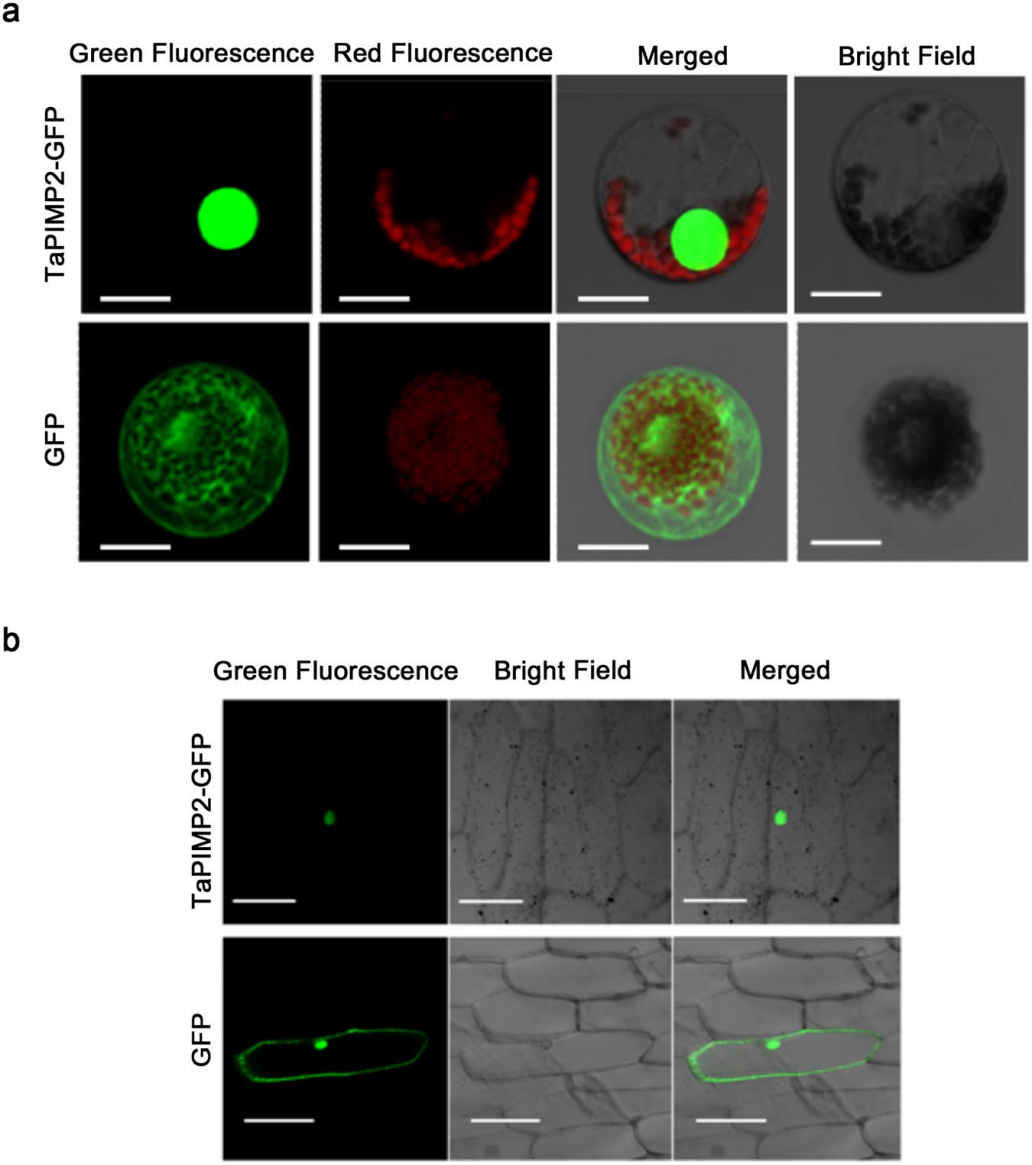
Subcellular localization of TaPIMP2 in wheat mesophyll protoplasts (**a**) and onion epidermal cells (**b**). TaPIMP2-GFP (green fluorescent protein) fusion protein is localized to nucleus. GFP alone is distributed in both cytoplasm and nucleus. The red fluorescence is from chloroplast autofluoresence. Images were captured using the following wavelengths, green fluorescence (excitation, 488 nm; emission, 509 nm) and red fluoresence (excitation, 448 nm; emission, 647 nm). Bars: 50 μm.

We investigated the subcelluar localization of TaPIMP2-1B in both wheat mesophyll protoplasts and onion epidermal cells. The p35S::TaPIMP2-GFP (green fluorescence protein) or control p35S::GFP construct was separately transiently expressed in wheat mesophyll protoplasts. As shown in Fig. 5a, the fluorescence signal of the TaPIMP2-GFP fusion protein was observed in nucleus of wheat mesophyll protoplasts, whereas the fluorescence of the control GFP was distributed in both cytoplasm and nucleus. As onion epidermal cells are lacked of interfering chlorophyll fluorescence, green fluorescence signals were readily observed. The p35S::TaPIMP2-GFP or control p35S::GFP construct was separately introduced into onion epidermal cells through particle bombardment. Confocal imaging of transient expression in the onion epidermal cells showed that TaPIMP2-GFP localized in the nucleus, whereas the fluorescence of the control GFP was distributed in both cytoplasm and nucleus (Fig. 5b). The above results proved that TaPIMP2 is localized and can be expressed in nucleus in both wheat mesophyll protoplasts and onion epidermal cells.

### *TaPIMP2* is required for wheat resistance to *B*. *sorokiniana*

**Figure 6:**
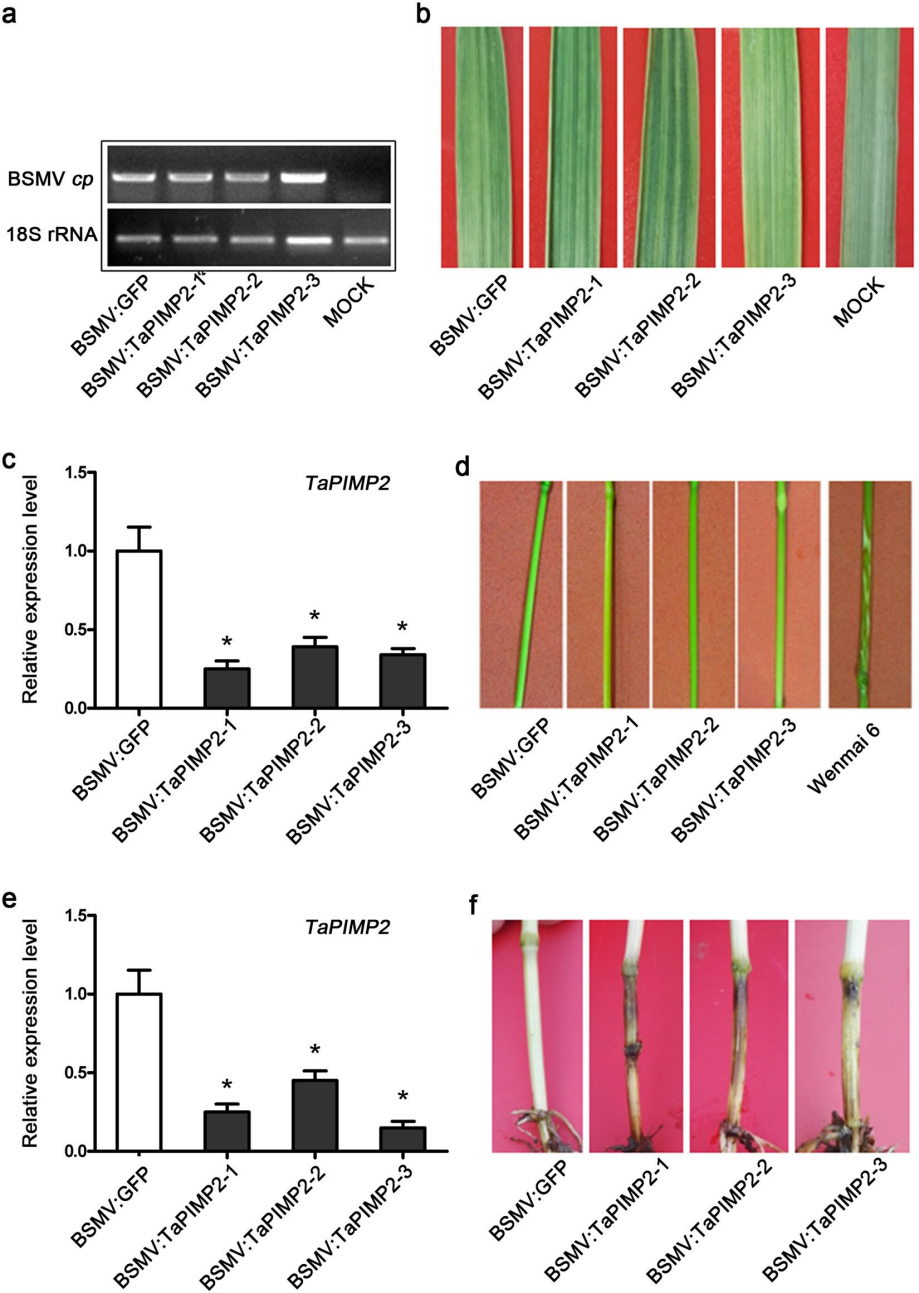
Responses of TaPIMP2-silenced wheat plants to infection with *Rhizoctonia cerealis* or *Bipolaris sorokiniana*. (**a**) The barley stripe mosaic virus coat protein (*cp*) gene was detected by RT-PCR. (**b**) Phenotypes of wheat leaves infected with BSMV:TaPIMP2 or BSMV:GFP (control). (**c**) Transcriptional analysis of *TaPIMP2* in stems of *TaPIMP2*-silencing CI12633 wheat and control plants. The expression level of *TaPIMP2* in control CI12633 plants was set to 1. (**d**) The typical sharp eyespot symptoms were displayed in the susceptible control wheat Wenmai 6. (**e**) Transcriptional analysis of *TaPIMP2* in TaPIMP2-silencing Yangmai 6 wheat and control plants. The expression level of *TaPIMP2* in control Yangmai 6 plants was set to 1. (**f**) The typical common root rot symptoms were displayed in the TaPIMP2-silencing Yangmai 6. Values represent the mean and standard deviation from three independent biological replicates (Student’s *t*-*test*: *P < 0.05).

As *TaPIMP2* transcription was induced after challenge with *R*. *cerealis* or *B*. *sorokiniana*, its defensive roles in wheat were investigated by barley stripe mosaic virus (BSMV)-mediated virus-induced gene silencing (VIGS). For the silencing purpose, a *TaPIMP2*-specific fragment (247 bp length, from 729 to 975 nt of cDNA sequence, spanning the 3′ terminal of ORF and 3′ untranslated region) was inserted in an antisense orientation into *Nhe* I restriction site of the BSMV γ strand to generate the recombinant vector **γ**:TaPIMP2. BSMV α, β, and γ or γ:GFP or recombinant **γ**:TaPIMP2 were transcribed and mixed to result in BSMV:GFP or BSMV:TaPIMP2 viruses. The BSMV:GFP or BSMV:TaPIMP2 viruses were inoculated onto leaves of 2 wheat lines (*R*. *cerealis*-resistant CI12633 and *B*. *sorokiniana*-resistant Yangmai 6). After 7 days of the virus inoculation, the expression of BSMV coat protein (CP) gene was readily detected in new emerged leaves (Fig. 6a), and the typical BSMV symptom was present in these tissues (Fig. 6b), proving that these inoculated plants were successfully infected by either BSMV:GFP or BSMV:TaPIMP2.

The silencing of *TaPIMP2* in stems of BSMV-infected wheat plants was analyzed by RT-qPCR. The conserved region sequence of *TaPIMP2*-*1A*, -*1B*, and -*1D* was used to design qPCR primers, which were used to detect simultaneously transcriptional levels of three *TaPIMP2* homoeologous members. The results showed that *TaPIMP2* transcriptional level was significantly decreased to 25~39% in BSMV:TaPIMP2-infected (TaPIMP2-silenced) CI12633 plants compared with BSMV:GFP-infected control plants (Fig. 6c). The *R*. *cerealis* isolate R0301 was inoculated on the stems of these BSMV-infected plants to evaluate the resistance role of *TaPIMP2* to sharp eyespot. Following inoculation with *R*. *cerealis* for 40 d, sharp eyespot symptoms did not obviously appear at inoculation site at both *TaPIMP2*-silencing and control wheat CI12633 plants, whereas serious sharp eyespot symptoms were presented in the *R*. *cerealis*-susceptible wheat cultivar Wenmai 6 (Fig. 6d). *B*. *sorokiniana*-resistance wheat cultivar Yangmai 6 plants were used for evaluating defense role of *TaPIMP2* to common root rot disease by VIGS. Compared with control plants, transcriptional level of *TaPIMP2* decreased to 21.5~32.2% in BSMV:TaPIMP2-infected Yangmai 6 plants (Fig. 6e). After inoculation with *B*. *sorokiniana* for 40 d, common root rot disease symptoms were heavily developed in TaPIMP2-silenced Yangmai 6, but BSMV:GFP-infected (control) Yangmai 6 plants exhibited weaker symptoms (Fig. 6f). The above results suggested that *TaPIMP2* was required for wheat defense to *B*. *sorokiniana* infection in Yangmai 6, but not required for wheat defense, at least in the resistant wheat line CI12633, to *R*. *cerealis*.

### Overexpression of *TaPIMP2* enhances wheat resistance to *B*. *sorokiniana*

**Figure 7:**
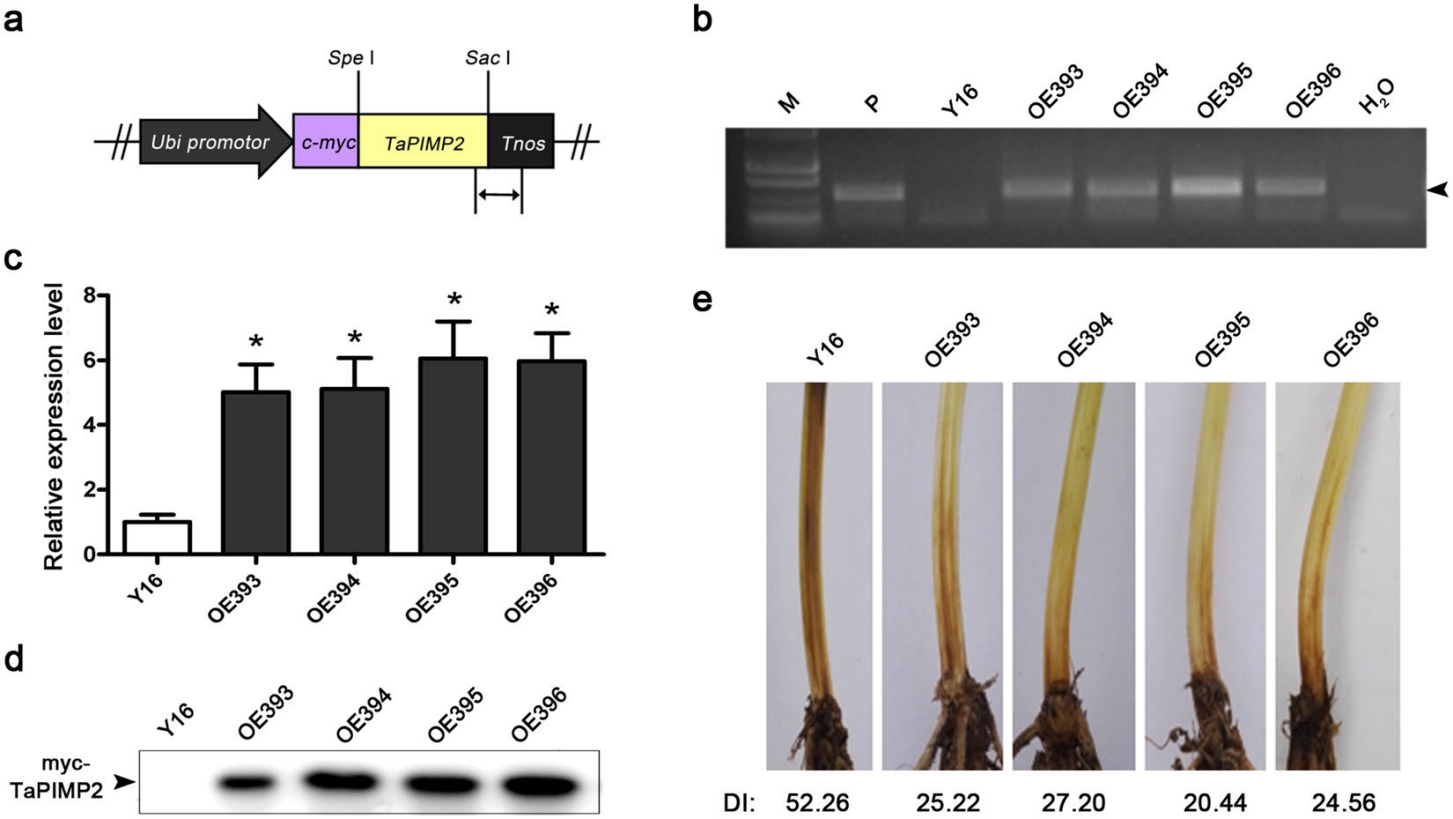
Molecular characterization of TaPIMP2-overexpression wheat plants and responses to *Bipolaris sorokiniana*. (**a**) Scheme of the TaPIMP2 expression cassette on the transformation vector pUBI::myc-TaPIMP2. *Ubi*: maize ubiquitin promoter; *Tnos*: terminator of Agrobacterium tumefaciens nopaline synthase gene. The arrow indicates the amplified regions of transgenic plants by PCR. (**b**) PCR patterns of the *TaPIMP2*-transgenic wheat lines using the primers specific to the TaPIMP2-Tnos cassette. M: molecular marker; P: plasmid pUBI::myc-TaPIMP2; Y16: the recipient Yangmai 16. (**c**) Relative expression levels of *TaPIMP2* in overexpressing wheat lines and untransformed Yangmai 16. The relative transcript level in the untransformed Yangmai 16 was set to 1. (**d**) Western blotting analysis of the TaPIMP2-overexpressing wheat lines and untransformed Yangmai 16 using an anti-myc antibody. (**e**) The typical common root rot symptoms of TaPIMP2-overexpression and untransformed Yangmai 16. Values represent the mean and standard deviation from three independent biological replicates (Student’s *t*-*test*: *P < 0.05).

To further explore the functional role in wheat defense response, *TaPIMP2*-overexpression wheat plants were generated. As the sequence of the target probe A_99_P129195 in Agilent Wheat GeneChip was corresponding to the homoeologous member *TaPIMP2*-*1B*, the *TaPIMP2*-*1B* was selected for transformation into wheat. The ORF sequence of *TaPIMP2*-*1B* was inserted into a modified monocot transformation vector pAHC25-myc^26^, resulting in the overexpression vector pUBI::myc-TaPIMP2 (Fig. 7a). In the transformation construct, the expression of the fused c-myc-TaPIMP2 of a c-myc epitope tag and *TaPIMP2*-*1B* was driven by a maize ubiquitin promoter (UBI) and terminated by the terminator of the *Agrobacterium tumefaciens* nopaline synthase gene (*Tnos*).

The pUBI::myc-TaPIMP2 plasmid was bombarded to immature embryos of the spring wheat cultivar Yangmai 16 for generating transgenic wheat plants. The presence of introduced *TaPIMP2* transgene was detected by the targeted PCR product (213 bp) using the primer pairs locating in TaPIMP2 and Tnos sequences (Fig. 7b), and the copy number in transgenic wheat plants was estimated by droplet digital PCR (ddPCR). The primers used in ddPCR assay were the same with those used in PCR detection for transgenic plants. The ddPCR has proven to be a reliable and reproducible method in estimation of transgene copy number in wheat^27^ and tobacco^28^. *TaSTOP1*-*A* was used as the reference with two copies in hexaploid wheat^29^. The data from ddPCR assay showed that copy numbers of introduced TaPIMP2-Tnos chimera were 8.210, 5.895, 8.145, and 11.206 in transgenic lines OE393, OE394, OE395, and OE396, respectively. Hence, the copy numbers of introduced TaPIMP2 gene were estimated as 8, 6, 8, and 11 in transgenic wheat lines OE393, OE394, OE395, and OE396, respectively.

Four transgenic lines containing TaPIMP2-Tnos (OE393, OE394, OE395, and OE396) were selected for disease resistance evaluation. RT-qPCR analysis showed that the transcript levels of *TaPIMP2* in stems from four overexpression transgenic lines were significantly elevated compared with untransformed (wild type, WT) Yangmai 16 (Fig. 7c). Using anti-c-myc as antibody, western blotting assay showed that the introduced c-myc-TaPIMP2 fusion protein was significantly accumulated in all four transgenic wheat lines, whereas no corresponding band was detected in untransformed Yangmai 16 (Fig. 7d). These results indicated that the introduced myc-TaPIMP2 was overexpressed and translated in these 4 transgenic wheat lines.

The defense responses of these 4 *TaPIMP2*-overexpression lines in T_4_ generation were evaluated following inoculating with *B*. *sorokiniana* or *R*. *cerealis*. Following inoculation with *B*. *sorokiniana* for 40 d, the disease severity scoring results showed that these four overexpression lines exhibited significantly enhanced resistance to common root rot compared to untransformed Yangmai 16. The average disease indices of the four overexpression lines were 25.22, 27.20, 20.44, and 24.56, respectively, whereas the disease index of untransformed Yangmai 16 was 52.26 (Fig. 7e). Furthermore, the responses of the four *TaPIMP2*-overexpression wheat lines to *R*. *cerealis* in T_1_-T_4_ generations were evaluated. The sharp eyespot infection types and disease indices did not exhibit significant differences between the four overexpressing wheat lines and untransformed wheat plants (Table S1). These results indicated that overexpression of *TaPIMP2* enhanced wheat resistance to infection of *B*. *sorokiniana* rather than *R*. *cerealis*.

### TaPIMP2 regulates transcription of pathogenesis-related genes in wheat

**Figure 8:**
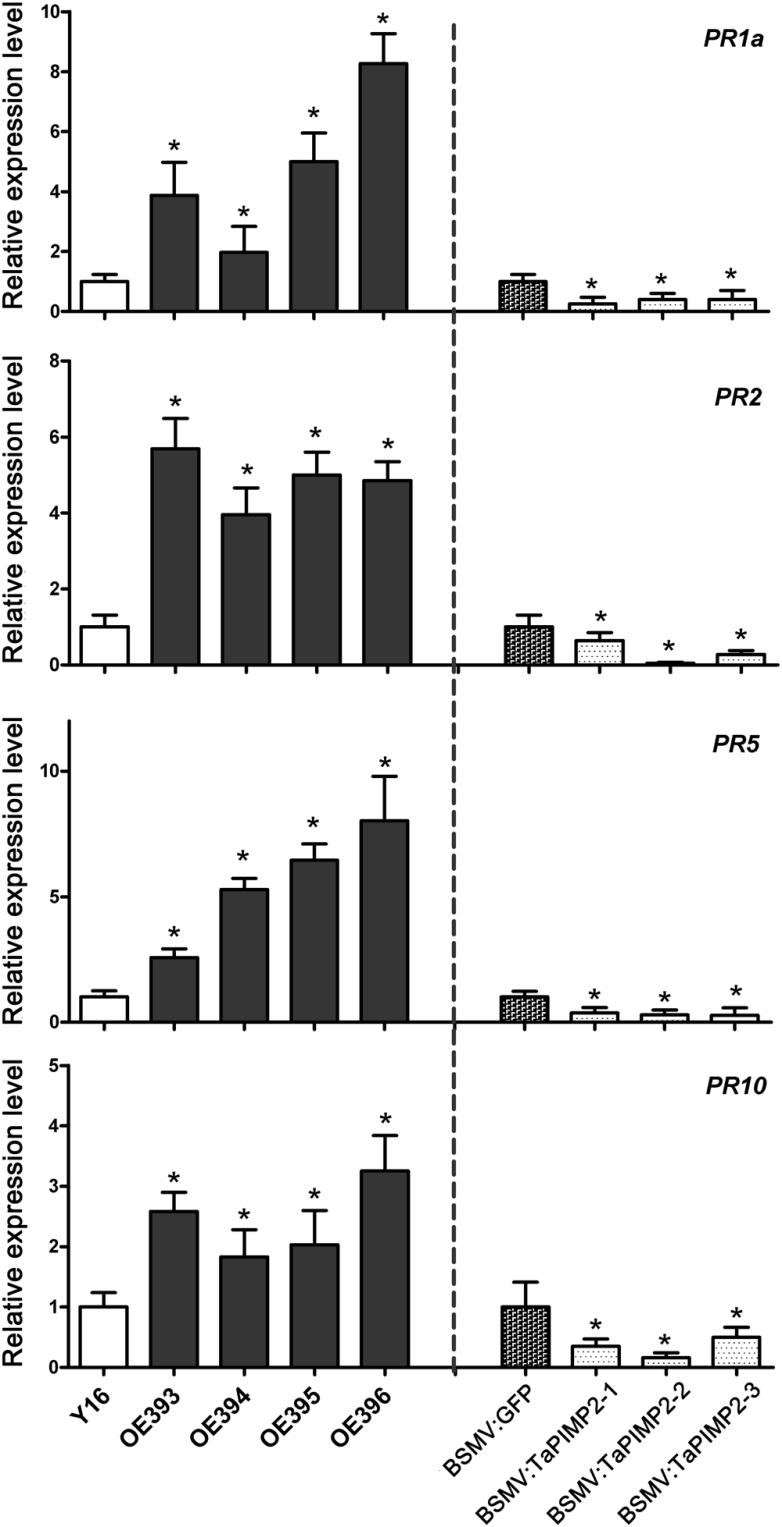
Expression of defense-associated genes in TaPIMP2-overexpressing wheat lines and TaPIMP2-silenced wheat plants by RT-qPCR. The transcript levels of these genes in TaPIMP2-overexpressing wheat lines are relative to those in untransformed Yangmai 16, whereas the levels in TaPIMP2-silenced wheat plants are relative to those in the control plants infected with BSMV:GFP. Values represent the average standard error of three independent biological replicates (Student’s t-test: *P < 0.05).

Pathogenesis-related (PR) proteins, including PR1a, PR2, PR5, and PR10, were shown to be involved in wheat resistance to *B*. *sorokiniana* or *R*. *cerealis* infection in previously publications^18, 30^. To explore if TaPIMP2 regulates the expression of PR genes, the above-mentioned PR protein-encoding genes were selected for transcriptional quantification analysis by RT-qPCR in *TaPIMP2*-overexpression wheat lines, *TaPIMP2*-silenced wheat plants, and their corresponding control wheat plants. RT-qPCR analysis results showed that the transcriptional levels of *PR1a*, *PR2*, *PR5*, and *PR10* were significantly increased in *TaPIMP2*-overexpression wheat lines compared with untransformed Yangmai 16, but significantly decreased in *TaPIMP2*-silenced wheat plants than that in BSMV:GFP-infected plants (Fig. 8). The results clearly revealed that *TaPIMP2* positively regulated the expression of certain defense-related genes in wheat.

## Discussion

In this study, *TaPIMP2*, a pathogen-induced MYB gene, was cloned from wheat based on comparative transcriptome. The deduced protein TaPIMP2 belongs to R2R3 MYB subgroup. Comparison of whole protein sequences showed that TaPIMP2 has no obvious sequence similarity to both TaPIMP1 and TaRIM1 except the MYB domains (Fig. 1). Previous studies showed that some MYB genes being involved in defense responses were induced after challenge with the pathogens. For example, a wheat R2R3-MYB gene *TaPIMP1* is induced after *B*. *sorokiniana* infection and positively regulates host defense response to the pathogen infection^18^. Both *TaMYB4* and *TaLHY* were induced after infection of *Pst* ^19, 20^. *TaRIM1* was induced upon infection with *R*. *cerealis* ^24^. Here, RT-qPCR analysis results indicated that infection of *R*. *cerealis* and *B*. *sorokiniana* triggered the transcriptional accumulation of *TaPIMP2* in wheat lines (Fig. 3a and b). However, the induced transcription patterns were different upon infection between the two pathogens. After infection of *R*. *cerealis*, *TaPIMP2* transcriptional level gradually increased at 1, 2, and 4 dpi, then sharply elevated and reached the peak (~14-fold) at 7 dpi (Fig. 3a). Upon challenging with *B*. *sorokiniana*, *TaPIMP2* transcript was rapidly and dramatically accumulated to ~60-fold at 1 dpi than that of untreated plants (Fig. 3b). The above results suggest that *TaPIMP2* showed a more sharp and rapid response to infection of *B*. *sorokiniana* than to *R*. *cerealis* infection.

Previous studies revealed that mutant/silencing of certain MYB genes (*AtMYB108*, *TaPIMP1*, *TaMYB4*, *TaLHY*, and *TaRIM1*) impaired resistance or enhanced susceptibility to different diseases, while overexpression of the MYB genes, such as *TaPIMP1* and *TaRIM1*, improved significantly resistance of transgenic plants to infection of pathogens^7, 18–20, 24^. In the present study, we dissected the functional roles of *TaPIMP2* to *B*. *sorokiniana* and *R*. *cerealis* through generation and assessment of *TaPIMP2* silencing and overexpression wheat plants. The results indicated that silencing of *TaPIMP2* significantly impaired wheat Yangmai 6 resistance to *B*. *sorokiniana*, but silencing of *TaPIMP2* did not obviously impair the resistance to *R*. *cerealis* in sharp eyespot-resistant wheat line CI12633 (Fig. 6). This discrepancy in responses to *R*. *cerealis* and *B*. *sorokiniana* in *TaPIMP2*-silenced wheat plants may be ascribed to that *TaPIMP2* could be involved in different defense pathways in CI12633 and Yangmai 6. Another possible reason may be that *TaPIMP2* is functionally redundant with other genes in CI12633 resistance response to *R*. *cerealis*. For example, in *Arabidopsis*, two MYB proteins MYB33 and MYB65 participate in anther development. However, neither single knockout mutant, *myb33* or *myb65*, display disturbed anther development^31^. Moreover, overexpression of *TaPIMP2* significantly increased resistance of transgenic wheat Yangmai 16 to common root rot caused by *B*. *sorokiniana*, but did not significantly alter resistance degree of the transgenic wheat lines to *R*. *cerealis* infection (Table S1). These results suggested that *TaPIMP2* positively contributes to resistance response to *B*. *sorokiniana*, but is not sufficient for improving disease resistance in Yangmai 16 to *R*. *cerealis*. Special MYB proteins may have multiple roles in different biotic and abiotic stresses^7, 8, 15, 18^. It is very interesting to further explore the role of TaPIMP2 in other areas including signal pathway.

Phytohormone, including SA, JA, ethylene, and ABA, as well as other small phytohormones, have been evidenced to play pivotal roles in immunity network^2, 32–34^. Usually, SA is associated with biotrophic pathogen resistance, whereas JA and ethylene are associated with necrotrophic pathogen resistance responses^2^. ABA plays a important role in regulation of defense responses to abiotic and biotic stresses^2, 33^. A number of TF families, e. g. ethylene-responsive factor (ERF) and MYB TFs, play important roles in the transmission of pathogen-derived defense signals. For instance, the Arabidopsis *BOS1* gene controls both JA- and ABA-inducible genes, consequently participates in defense responses to necrotrophic pathogens and abiotic stresses^7^. AtMYB96-mediated ABA signaling promotes drought tolerance and resistance to the pathogen *Pseudomonas syringae* pv. tomato DC3000 infection by inducing SA biosynthesis^8, 15^. In *Arabidopsis*, cooperation of JA and ethylene in activation of defense against necrotrophic pathogens can be explained by the activation of the nodes ERF1 or ORA59, two members of the AP2/ERF transcription factor subfamily^35, 36^. Previously, our lab studies revealed that the wheat MYB TF TaPIMP1, being up-regulated by exogenous ABA and SA positively regulates host resistance to *B*. *sorokiniana* and drought through ABA and SA signaling pathways^18^; and a wheat ERF TF *TaPIE1* being induced by ethylene positively contributes to resistance to *R*. *cerealis*, and freezing stresses mainly through the ethylene signaling pathway^30^. In this study, the transcription of *TaPIMP2* was rapidly and strongly induced by ABA, while was transiently induced by ACC, the precursor of ethylene (Fig. 4). These data suggested that TaPIMP2 might be modulated in a distinct extent through ABA and ethylene signal pathways in defense responses to *B*. *sorokiniana* and *R*. *cerealis*. The underlying mechanism needs to be explored in future.

Various PR proteins played important roles in plant defense responses to different pathogen infection^37–39^. In this study, we analyzed the transcriptional levels of four selected PR genes (including *PR1a*, *PR2*, *PR5*, and *PR10*) in *TaPIMP2*-overexpression wheat lines, *TaPIMP2*-silencing wheat plants, and their corresponding control wheat plants (Fig. 8). The results revealed that TaPIMP2 positively modulated the expression of the tested PR genes. Increasing evidence indicates that transcription factors may modulate the expression of defense-related genes through binding to corresponding *cis*-acting elements. For example, the wheat MYB TF TaPIMP1 can activate the expression of 5 defense-related genes by binding to the MYB-binding sequence (MBS) *cis*-elements in their promoters^18^. An MYB TF BjMYB1 from *Brassica juncea* could active the expression of a chitinase gene *BjCHI1* through binding to the W-box element WbI-4 in its promoter^40^. The upland cotton MYB TF GhMYB108 could bind to the MBS *cis*-element^41^. Previous studies showed that the promoter regions of *PR1a* and *PR10* contain ACI and MBS *cis*-elements^18, 24^. Our analyses on the promoter regions of *PR2* and *PR5* revealed that 2 MYB-binding sequences (ACI and MBS) and 1 W-box *cis*-element were present in these promoters (Table S2). These data suggested that TaPIMP2 might bind to MBS *cis*-elements in these promoters of the tested PR genes and active directly or indirectly their expression, leading to enhanced resistance in overexpression transgenic wheat.

In conclusion, *TaPIMP2*, a pathogen-induce MYB protein-encoding gene in wheat, was isolated and its defense function against *R*. *cerealis* or *B*. *sorokiniana* was characterized. Transcriptional level of *TaPIMP2* was rapidly and strongly increased after treatments with *B*. *sorokiniana* and ABA, while was moderately induced by *R*. *cerealis* and transiently by ethylene, or depressed by SA, respectively. *TaPIMP2* positively regulates wheat resistance defense to common root rot caused by *B*. *sorokiniana* through modulating transcription of certain PR genes. Our study provided new insight into functional diversity of MYB TFs in plant defense responses to infection of different pathogens.

## Materials and Methods

### Plant and fungal materials, and treatments

Four wheat lines/cultivars, CI12633, Wenmai 6, Yangmai 6, and Yangmai 16 were used in this study. Among them, CI12633 is resistant to *R*. *cerealis* infection, whereas Wenmai 6 is highly susceptible. Yangmai 6 is resistant to infection of *B*. *sorokiniana*. Yangmai 16 is moderately susceptible to both *R*. *cerealis* and *B*. *sorokiniana* infection, and used as transgenic recipient. The pathogenic fungus *R*. *cerealis* isolate R0301 was provided by Profs. Huaigu Chen and Shibin Cai from Jiangsu Academy of Agricultural Sciences, China. *B*. *sorokiniana* isolate Hn was provided by Prof. Hongjie Li from Institute of Crop Science, Chinese Academy of Agricultural Sciences.

Wheat plants were grown in a 14 h light/10 h dark (22 °C/10 °C) regime. The wheat plants inoculation with *R*. *cerealis* was followed the method of Chen *et al*.^23^. Briefly, the soaked wheat kernels were autoclaved for 20 min at 121 °C and then inoculated with fungal discs cut at the edge of freshly cultured *R*. *cerealis* on patato dextrose agar plate. The inoculated kernels were incubated for 14 days at 25 °C. The *R*. *cerealis*-colonized wheat kernels were placed on the soil surface in contact with the stem base of wheat plants. The inoculation of *B*. *sorokiniana* was conducted followed by Zhang *et al*. procedures^18^. Briefly, the *B*. *sorokiniana*-colonized wheat kernels were placed on the soil surface in contact with the stem base of wheat plants.

The wheat cultivar Yangmai 16 plants at the three-leaf stage were sprayed with 1.0 mM ABA, 50 μM ACC, 1.0 mM SA, and 0.1% Tween-20 solution. Wheat plants treated with Tween-20 solution were used as mock control. The wheat leaves were sampled for detecting the transcription of *TaPIMP2*.

### RNA extraction and cDNA synthesis

Total RNA was extracted using TRIZOL reagent (Invitrogen) and subjected to DNase I (TaKaRa) digestion and purification. The first-strand cDNA was synthesized using 2-μg purified RNA, AMV reverse transcriptase and oligo(dT_15_) primers (TaKaRa) according to the product instruction.

### Cloning of full-length sequence of *TaPIMP2* and sequences analyses

Full-length cDNA sequence of *TaPIMP2* was obtained from CI12633 through 3′-RACE core set kit (TaKaRa) and RT-PCR. The full-length of cDNA and genomic DNA of *TaPIMP2* were obtained from CI12633 using the primers TaPIMP2-fl-F and TaPIMP2-fl-R. The purified PCR products were cloned into the pMD-18T vector (TaKaRa) and sequenced. The cDNA and genomic DNA was analyzed using BLAST (http://blast.ncbi.nlm.nih.gov/Blast.cgi). Pfam software (http://pfam.xfam.org/) was used for prediction of the conserved domains and motifs. A phylogenetic tree was constructed by the neighbor-joining method using MEGA 5.0 software. The amino acid sequence alignment of MYB proteins was analyzed by Clustal X software.

### Subcellular localization of TaPIMP2 in wheat and onion cells

The coding region of TaPIMP2 lacking the stop codon was amplified using the primers TaPIMP2-GFP_F and TaPIMP2-GFP_R containing restriction sites *Sal I* and *BamH I*, respectively. The PCR products was digested with restriction enzymes *Sal* I and *Bam*H I, then subcloned in-frame into the 5-terminus of the green fluorescent protein (GFP) coding region in the p35S::GFP vector (kindly provided by Dr. DaowenWang, Chinese Academy of Sciences), resulting in the TaPIMP2-GFP fusion construct p35S::TaPIMP2-GFP.

The p35S::TaPIMP2-GFP or p35S::GFP vector construct was separately transformed into onion epidermal cell by particle bombardment. For wheat protoplast assay, the TaPIMP2-GFP fusion or GFP alone construct was separately introduced into wheat protoplasts via the PEG-mediated transfection method following Yoo *et al*.^42^. The transformed onion epidermal cells or wheat protoplasts cells were incubated at 25 °C for 15 h. The GFP signals were observed and photographed using a Confocal Laser Scanning Microscopy (Zeiss LSM 700, Germany) with a Fluar 10X/0.50 M27 objective lens and SP640 filter.

### Real-time quantitative RT-PCR (RT-qPCR) analyses of *TaPIMP2* and defense-related genes

For RT-qPCR assay, the stems of wheat plants were sampled for examining the transcriptional level of target genes in TaPIMP2-silencing plants and –overexpression wheat plants. RT-qPCR was performed using SYBR Green I Master Mix (TaKaRa) using ABI PRISM 7500 detective system according to the manufacturer’s instruction. The primers of RT-qPCR for *TaPIMP2* homoeologous members and 4 defense-related genes were listed in the Table 1. RT-qPCR reaction was set up with the following thermal profile: 95 °C for 5 min, 41 cycles of 95 °C for 15 s and 60 °C for 31 s. The relative transcript levels of tested genes were calculated using the 2^−ΔΔCT^ method^43^, where the wheat *actin* gene (Genbank accession no. BE425627) was used as reference.

**Table 1:** The primers used in this study.

Primer	Sequence (5′ to 3′)	Usage	Reference
TaPIMP2-fl_F	TGCCTAGCTCGTGGGAGTAG	Full-length cloning of *TaPIMP2*	This study
TaPIMP2-fl_R	CAATGGTTCTTTGCTGTCCTG		
TaPIMP2-GFP_F	ATGTCGACATGGGACGTCCGTCGTC	Construction of subcellular localization vector	This study
TaPIMP2-GFP_R	GTCGGATCCGAAGTATGGTTCCAATTCC		
TaPIMP2-NheIF	CATGCTAGCCTCCGAGAATCTGGGCTACG	Construction of VIGS vector	This study
TaPIMP2-NheIR	CTGCTAGCCCGGGGAGAAAGAAAGAAGA		
TaPIMP2-OE_F	ATACTAGTATGGGACGTCCGTCGTC	Construction of transformation vector	This study
TaPIMP2-OE_R	GTCGAGCTCGAAGTATGGTTCCAATTCC		
TaPIMP2-TRANS_F	TTTTGATTTCAACTTGGAATTGG	PCR detection and ddPCR of introduced TaPIMP2	This study
TNOS-R	ATGTATAATTGCGGGACTCTAAT		
TaSTOP1A-F	GCAGAGGAGCGAGGCGATGGACGAC	ddPCR of reference TaSTOP1-A	This study
TaSTOP1A-R	GCTGCAAGAACCCGGTCC TGAAG		
TaPIMP2-1A_qF	AACAGAGCCTCCTCGCAAGT	Quantification of *TaPIMP2*-*1A*	This study
TaPIMP2-1A_qR	AAGAAGGTAAAAAATGGAGGGAA		
TaPIMP2-1B_qF	AACAGAGCCTCCTCGCAAGT	Quantification of *TaPIMP2*-*1B*	This study
TaPIMP2-1B_qR	GGTGGGAATCGAGATAATTGG		
TaPIMP2-1D_qF	CCGCTAGAGCACAAGTTGTCG	Quantification of *TaPIMP2*-*1D*	This study
TaPIMP2-1D_qR	GGGTGGGAATCGAGATAATTCA		
TaPIMP2-qF	GCATTGTACGGCCAGTTCG	Quantification of *TaPIMP2*	This study
TaPIMP2-qR	CGAGGAGGCTCTGTTCTTGG		
TaACTIN-qF	CACTGGAATGGTCAAGGCTG	Quantification of *TaACTIN*	Zhang *et al*.^18^
TaACTIN-qR	CTCCATGTCATCCCAGTTG		
PR1a-qF	CGTCTTCATCACCTGCAACTA	Quantification of *PR1a*	Zhang *et al*.^18^
PR1a-qR	CAAACATAAACACACGCACGTA		
PR2-qF	CCGCACAAGACACCTCAAGATA	Quantification of *PR2*	Zhang *et al*.^18^
PR2-qR	CGATGCCCTTGGTTTGGTAGA		
PR5-qF	ACAGCTACGCCAAGGACGAC	Quantification of *PR5*	Ameya *et al*.^39^
PR5-qR	CGCGTCCTAATCTAAGGGCAG		
PR10-qF	CGTGGAGGTAAACGATGAG	Quantification of *PR10*	Zhu *et al*.^25^
PR10-qR	GCTAAGTGTCCGGGGTAAT		

### Functional analysis of *TaPIMP2* through VIGS

To generate BSMV:TaPIMP2 construct, TaPIMP2-specific cDNA fragment was amplified by primers TaPIMP2-NheIF and TaPIMP2-NheIF containing *Nhe* I restriction site from cDNA of CI12633. PCR products were digested with *Nhe* I, and then ligated into the BSMV-γ vector, resulting in recombinant vector BSMV-γ-TaPIMP2.

The virus BSMV:TaPIMP2 and control virus BSMV:GFP were used to inoculate the CI12633 or Yangmai 6 following Wang *et al*.^44^. The stems of BSMV-infected wheat plants were sampled for detection of expression of *TaPIMP2*. At 20 day after BSMV infection, the fungus *R*. *cerealis* or *B*. *sorokiniana* was used to inoculate the BSMV infected plants following the methods mentioned above. Disease symptom was observed at 40 day after inoculation with *R*. *cerealis* ^25^ or *B*. *sorokiniana* ^18^.

### *TaPIMP2*-overexpressing transformation vector construction and transformation into wheat

The *TaPIMP2* ORF sequence with the *Spe* I and *Sac* I restriction sites was amplified and then sub-cloned into the *Spe* I and *Sac* I sites of a modified monocot transformation vector pAHC25::myc^26^. According to the protocol described by Zhu *et al*.^25^, the plasmid DNA of resulting overexpression transformation vector pUBI::myc-TaPIMP2 was transformed into immature embryos of the wheat cultivar Yangmai 16 by biolistic bombardment.

### PCR and western blotting analyses on *TaPIMP2*-overexpressing transgenic wheat

The presence of the introduced *TaPIMP2* in the transformed wheat plants was monitored by PCR using the primers TaPIMP2-TRANS_F and Tnos-R that specific to TaPIMP2-Tnos cassette. The nontransformed Yangmai 16 and pUBI::myc-TaPIMP2 plasmid DNA were used as negative control and positive control, respectively. The PCR reaction was performed in a total volume of 20 μl containing 1 × *Taq* buffer, 1.5 mM Mg^2+^, 0.05 mM dNTP each; 0.4 mM each primer, 1 Unit *Taq* polymerase (TaKaRa), and 50 ng template DNA, with an initial denaturation at 94 °C 3 min, followed by 35 cycles of 94 °C 45 s, 54 °C 45 s and 72 °C 45 s, and a final extension at 72 °C 10 min. The desired PCR product (213 bp) specific to the introduced *TaPIMP2*-*Tnos* cassette was resolved on a 1.5% agarose gel and visualized by ethidium bromide staining.

The c-myc-TaPIMP2 fusion protein in the overexpressing transgenic wheat lines was detected by western blotting analysis. Total proteins were extracted from ~0.5 g inoculated stems and sheaths. Total soluble proteins (~10 μg) for each line were separated on 12% SDS-PAGE and transferred to polyvinyl difluoride membranes (Amersham). The blotting membranes were incubated with 900-fold diluted Anti-c-myc Mouse Monoclonal Antibody (Transgen Biotech) at 4 °C overnight, then incubated with 1000-fold diluted Goat Anti-Mouse IgG (H + L), HPR conjugated secondary antibody (Transgen Biotech) at 22 °C for 1 h. The c-myc-TaPIMP2 proteins were visualized using the Pro-light HRP Chemiluminescent Kit (Tiangen Biotech).

### The copy number estimation of introduced *TaPIMP2*-*1B* gene in transgenic wheat lines

DNA for ddPCR assay was digested by *Sal* I (TaKaRa). The digested DNA concentration was diluted to 40 ng/μl. For ddPCR reaction, each 20 μl PCR reaction contained 300 ng of DNA template, 100 nmol of each forward primer TaPIMP2-TRANS-Rand reverse primers Tnos-R, and 10 μl EvaGreen Supermix (Bio-Rad). The TaSTOP1-A (Genbank No: KF034796) was used as reference with two copies in hexaploid wheat. The primer TaSTOP1A-F and −R were targeted the reference. Droplets were generated by a QX200 droplet generator (Bio-Rad). A T100 thermal cycler (Bio-Rad) was used to run PCR using the following program: 1 cycle at 95 °C for 10 min, 45 cycles of denaturation at 95 °C for 30 s, annealing and extension at 60 °C for 1 min. Droplets were read using a QX200 droplet reader and analyzed with Quantasoft software (Bio-Rad).

### Assessment on responses of transgenic and recipient wheat plants to *B*. *sorokiniana* and *R*. *cerealis*

Thirty plants for each line of TaPIMP2-overexpressors in T_4_ generations and untransformed Yangmai 16 wheat were evaluated responses to *R*. *cerealis* or *B*. *sorokiniana*. *R*. *cerealis* disease evaluation were followed the method of Zhu *et al*.^25^. Disease infection type (IT) of sharp eyespot was scored based on the 0–5 disease scale: 0: no symptoms observed 1: lesions appeared on the sheaths rather than stems; 2: lesions covered less than 1/2 of infected stem perimeter; 3: lesions covered 1/2–3/4 of infected stem perimeter; 4: lesions covered more than 3/4; 5: dead plant. Disease index

$$\begin{array}{rcl}({\rm{DI}}) & = & \{(0\times {{\rm{X}}}_{0}+1\times {{\rm{X}}}_{1}+2\times {{\rm{X}}}_{2}+3\times {{\rm{X}}}_{3}+4\times {{\rm{X}}}_{4}+5\times {{\rm{X}}}_{5})\\  &  & /[({{\rm{X}}}_{0}+{{\rm{X}}}_{1}+{{\rm{X}}}_{2}\,+\,{{\rm{X}}}_{3}+{{\rm{X}}}_{4}+{{\rm{X}}}_{5})\times 5]\}\times 100,\end{array}$$

where X_0_–X_5_ indicated plants with IT: 0–5. *B*. *sorokiniana* inoculation and disease evaluation were followed protocols of Zhang *et al*.^18^. Disease infection type (IT) of common root rot was categorized from 0 to 4 based on the brown lesion square on the plant stem base. 0: no necrotic lision; 1: lesions covered less than 1/4 of infected stem perimeter; 2: lesions covered 1/4–1/2; 3: lesion covered 1/2–2/3; 4: lesions covered 2/3 to complete stem base. The disease index

$$\begin{array}{rcl}({\rm{DI}}) & = & \{(0\times {{\rm{X}}}_{0}+1\times {{\rm{X}}}_{1}+2\times {{\rm{X}}}_{2}+3\times {{\rm{X}}}_{3}+4\times {{\rm{X}}}_{4})/[({{\rm{X}}}_{0}+{{\rm{X}}}_{1}+{{\rm{X}}}_{2}\\  &  & +{{\rm{X}}}_{3}+{{\rm{X}}}_{4})\times 4]\}\times 100,\end{array}$$

where X_0_–X_4_ indicated plants with IT: 0–4^31^.

## Electronic supplementary material

Supplementary file
