# Supplementary material for: TaPIMP2, a pathogen-induced MYB protein in wheat, contributes to host resistance to common root rot caused by Bipolaris sorokiniana
Source: Sci Rep. 2017 May 11;7:1754. doi: 10.1038/s41598-017-01918-7 (PMC5431884; doi:10.1038/s41598-017-01918-7)
Supplement: Supplementary file 1 — Supplementary file [file 41598_2017_1918_MOESM1_ESM.pdf]

Title: TaPIMP2, a pathogen-induced MYB protein in wheat, contributes to host resistance to common root rot caused by *Bipolaris sorokiniana*

Authors: Xuening Wei†, Tianlei Shan†, Yantao Hong†, Huijun Xu, Xin Liu, Zengyan Zhang*

Supplementary table 1 *Rhizoctonia cerealis* responses of four TaPIMP2-overexpression Yangmai 16 transgenic lines and untransformed Yangmai 16.

Lines	ITs (infection tyeps)	DI (disease index)
OE393	2.12	42.40
OE394	2.08	41.60
OE395	2.06	41.20
OE396	2.40	48.00
Yangmai 16	2.17	43.40

Disease infection type (IT) of sharp eyespot was scored based on the 0-5 disease scale: 0: no symptoms observed 1: lesions appeared on the sheaths rather than stems; 2: lesions covered less than 1/2 of infected stem perimeter; 3: lesions covered 1/2-3/4 of infected stem perimeter; 4: lesions covered more than 3/4; 5: dead plant. Disease index $=\{(0\times X_0+1\times X_l+2\times X_2+3\times X_3+4\times X_4+5\times X_5)/[(X_0+X_1+X_2+X_3+X_4+X_5)\times 5]\}\times 100$, where X_0−X_5 indicated plants with IT: 0−5.

Supplementary table 2 MBS sequences in promoters of defense-related genes, *PR2* and *PR5*, regulated by TaPIMP2.

Gene name	Accession no.	Site name	Position	sequences
PR2	AF112965	MBS	701	CGGTCA
		ACI	1068	CACCCTCACC
PR5	AF442967	ACI	382	CCCACCTACC
		W-box	787	TTGACC

The sequence of 2500 bp upstream of ATG start codon were analyzed in PlantCARE (http://bioinformatics.psb.ugent.be/webtools/plantcare/html/).
